# TLR4 Expression by Liver Resident Cells Mediates the Development of Glucose Intolerance and Insulin Resistance in Experimental Periodontitis

**DOI:** 10.1371/journal.pone.0136502

**Published:** 2015-08-28

**Authors:** Vladimir Ilievski, Yale Cho, Priya Katwala, Heriberto Rodriguez, Margaret Tulowiecka, David Kurian, Lara Leoni, John W. Christman, Terry G. Unterman, Keiko Watanabe

**Affiliations:** 1 Department of Periodontics, College of Dentistry, University of Illinois at Chicago, Chicago, Illinois, United States of America; 2 Undergraduate Program, College of Dentistry, University of Illinois at Chicago, Chicago, Illinois, United States of America; 3 Undergraduate Program, University of Illinois at Chicago, Chicago, Illinois, United States of America; 4 Department of Radiology, University of Chicago, Chicago, Illinois, United States of America; 5 Department of Internal Medicine, Section of Pulmonary, Allergy, Critical Care, and Sleep Medicine, The Ohio State University, Columbus, Ohio, United States of America; 6 Departments of Medicine and Physiology and Biophysics, College of Medicine, University of Illinois at Chicago, and Jesse Brown VA Medical Center, Chicago, Illinois, United States of America; University of São Paulo, BRAZIL

## Abstract

**Background:**

Results from epidemiological studies indicate a close association between periodontitis and type 2 diabetes mellitus. However, the mechanism linking periodontitis to glucose intolerance (GI) and insulin resistance (IR) is unknown. We therefore tested the hypothesis that periodontitis induces the development of GI/IR through a liver Toll-like receptor 4 (TLR4) dependent mechanism.

**Methods:**

TLR4 chimeric mice were developed by bone marrow transplantation using green fluorescent protein expressing TLR4WT mouse (GFPWT) as donor and TLR4 WT or TLR4-/- as recipient mice (GFPWT:WT and GFPWT:KO chimeras respectively). These chimeras were subjected to experimental chronic periodontitis induced by repeated applications of LPS to the gingival sulci for 18 weeks. The levels of GI/IR were monitored and plasma cytokines and LPS were determined at 18 weeks when differences in glucose tolerance were most apparent. Cytokine gene expression was measured in liver tissue by qPCR.

**Results:**

Alveolar bone loss was significantly greater in GFPWT:WT chimeras treated with LPS compared with chimeras treated with PBS or GFPWT:KO chimeras. However, the degree of gingival inflammation was similar between GFPWT:WT and GFPWT:KO mice with LPS application. Severe GI/IR occurred in GFPWT:WT chimeras but not in the GFPWT:KO chimeras that were subjected to 18 weeks of LPS. Serum LPS was detected only in animals to which LPS was applied and the level was similar in GFPWT:WT and GFPWT:KO mice at the 18 week time point. Surprisingly, there was no significant difference in the plasma levels of IL1β, IL6 and TNFα at 18 weeks in spite of the severe GI/IR in the GFPWT:WT chimeras with LPS application. Also, no difference in the expression of TNFα or IL6 mRNA was detected in the liver of GFPWT:WT vs GFPWT:KO mice. In contrast, liver IL1β expression was significantly greater in GFPWT:WT chimeras compared to GFPWT:KO chimeras treated with LPS.

**Conclusion:**

We observed that GFPWT:WT, but not GFPWT:KO chimeras, treated with LPS developed GI/IR despite similar degrees of gingival inflammation, circulating cytokine levels, and LPS concentrations. We conclude that LPS from periodontitis sites has a pivotal role in triggering the development of GI/IR through a mechanism that involves TLR4 expression by resident macrophages/Kupffer cells in the liver.

## Introduction

Periodontitis is a disease characterized by destruction of gingiva and tooth-supporting bone caused by an exuberant host immunological response to periodontal pathogens or their byproducts such as lipopolysaccharide (LPS). In the absence of treatment, periodontitis leads to tooth loss. It is estimated that approximately 50% of adults in the US suffer from periodontitis [1], and is therefore an important public health problem.

Results from many epidemiological studies indicate a close association between periodontitis and type 2 diabetes mellitus (T2DM) and this relationship appears to be bidirectional [2,3]. Similarly, an association between periodontitis and prediabetes, i.e. glucose intolerance (GI) and insulin resistance (IR), is suggested by results from several epidemiological studies [4–6]. However, the mechanism(s) by which periodontitis impacts the development of GI/IR remain unclear. Preventing the development of the prediabetic condition is an important public health concern since over 35% of people are estimated to have prediabetes in the US [7]. The identification of risk factors for prediabetes and their mechanisms would be important contributions to public health since the prediabetic condition leads to T2DM with possible diabetic complications including heart attack, stroke, diabetic retinopathy, impaired wound healing, diabetic nephropathy, and atherosclerosis [8]. These diseases affect not only life expectancy and quality of life, but also the treatment of these complications involves extensive drug therapy and their associated exorbitant costs [9]. The results from recent studies indicate that diabetic complications are already initiated in the prediabetic condition [10–13]. Thus, if periodontitis induces prediabetes, then it must be considered a risk factor for the development of T2DM and its associated complications.

It is commonly suggested that periodontitis influences systemic health through the activities of cytokines and/or LPS that originate in the gingiva and enter the systemic circulation thereby reaching target organs to cause insulin signaling impairment, i.e. insulin resistance (IR) [14,15]. However, the precise pathways and the downstream effects of periodontitis on the development of prediabetes have not been definitively identified.

We have previously shown that periodontitis influences the course of diabetes development by accelerating the onset of IR, prediabetes, and T2DM in animal models [16]. We have also shown that periodontitis induced by placement of LPS soaked ligatures around molar teeth impairs hepatic insulin signaling via TLR4 and this impairment is reduced in mice with whole body TLR4 loss of function (LOF) in animals fed a high fat (HF) diet [17]. In addition, the extent of periodontitis based on the amount of alveolar bone loss is less in TLR4 LOF animals compared to that of wild type (WT). However, the specifics of how periodontitis promotes IR are not clear. It is known that macrophages express high levels of TLR 4 and activation of TLR4 leads to production of proinflammatory cytokines such as TNFα and IL1β [18]. These proinflammatory cytokines can, in turn, impair hepatic insulin signal transduction [19,20]. Thus, we propose that insulin signaling may be improved in mice with TLR LOF because periodontitis is less severe in these animals. This may result in lower cytokine production in the periodontitis sites resulting in decreased levels of cytokines in the systemic circulation compared with TLR4 WT mice. Alternatively, liver Kupffer cells (liver resident/tissue macrophages) in mice with TLR4 LOF do not respond to LPS originating from periodontitis sites so that the TLR4-induced cytokine response in the liver is blocked.

The difficulty in differentiating between these two possible mechanisms is that macrophages in the two locations (periodontium and liver) both express TLR4 and can be simultaneously activated by a TLR4 agonist in WT animals. Alternatively, macrophages in both locations may be non-activated in animals with TLR4 LOF. Thus, in order to differentiate between the two proposed mechanisms, we developed a model in which the phenotype of TLR4 differs between the two sites in the presence of chronic periodontitis.

The primary objective of this study was to test the hypothesis that periodontitis can cause prediabetes (GI and IR) and the primary pathway that leads to GI/IR is dependent on the phenotype of TLR4 expressed by the resident cells in the liver, primarily Kupffer cells. To test this hypothesis, we created an animal model that expresses WT-TLR4 (TLR4+/+) in macrophages in the gingiva, but whose liver resident cells are either TLR4+/+ (GFPWT:WT chimeras) or TLR4-/- (GFPWT:TLR4-/- chimeras referred to as GFPWT:KO) by performing adoptive bone marrow transplantation and inducing periodontitis.

**Fig 1:**
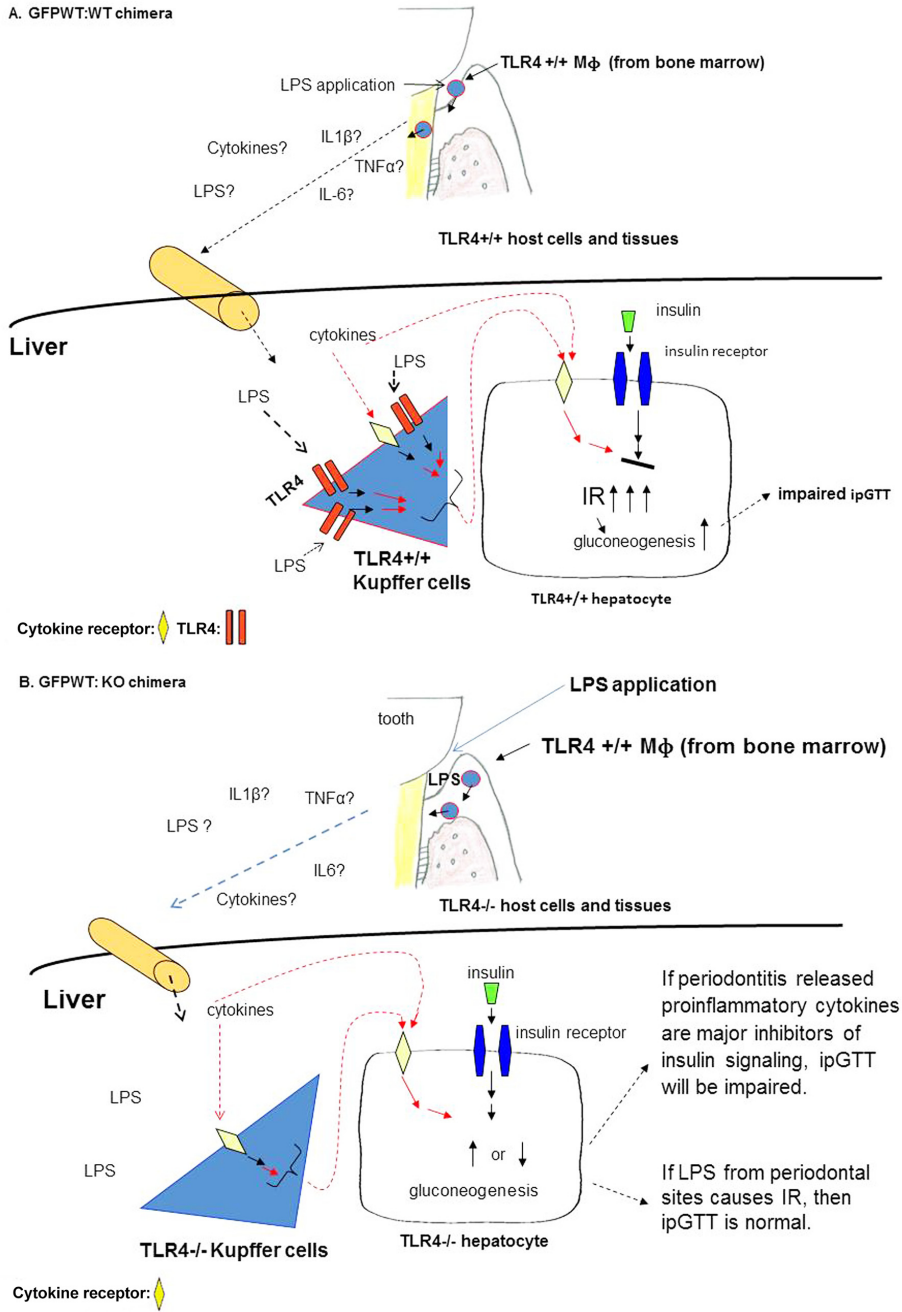
Hypothetical impact of LPS from the periodontium on the liver following LPS application to the gingival sulcus and the effects on glucose tolerance. Pathways represent the proposed activity in the periodontium and in the liver in GFPWT:WT (A) and GFP-WT:KO chimeras (B) following LPS application to the gingival sulcus. (A) LPS from gram negative bacteria is released in the gingival sulci where it acts as a chemoattractant. Macrophages (TLR4+/+) from circulating blood migrate to the gingival tissues where they are activated by LPS and secrete proinflammatory cytokines such as TNFα, IL1β and IL6. These cytokines and/or LPS enter the circulation and eventually reach the liver. Liver Kupffer cells that reside in sinusoids will be exposed to either LPS and/or proinflammatory cytokines. In GFPWT:WT animals Kupffer cells are TLR4+/+ and will respond to LPS by synthesizing and secreting proinflammatory cytokines resulting in impaired ipGTT. In addition, cytokines from the circulation can impair insulin signaling pathway in hepatocytes. (B) In GFPWT:KO animals, Kupffer cells are TLR4-/- and if the major stimulus to the liver Kupffer cells is LPS, ipGTT in these animals will be normal. However, circulating proinflammatory cytokines may act directly on hepatocytes leading to impaired ipGTT. IR: insulin resistance, ipGTT: intraperitoneal glucose tolerance test.

A commonly used procedure to generate chimeric mice by adoptive bone marrow transplantation is to irradiate a recipient mouse to eliminate bone marrow and circulating cells in conjunction with the use of clodronate liposomes which eradicate tissue phagocytic cells, primarily liver Kupffer cells. In this study, we did not use clodronate in order to retain TLR4-/- Kupffer cells in GFPWT:KO chimeras. Our basic premise is that if the primary trigger for IR is circulating cytokines, then GI/IR will be impaired in both GFPWT:WT and GFPWT:KO chimeras, since these cytokines would cause impaired glucose tolerance without activation of liver TLR4. However, if the primary trigger is circulating LPS, then GFPWT:WT chimeras with periodontitis will develop impaired GI/IR, but the GFPWT:KO chimeras with periodontitis will not, as the liver tissue resident macrophages (primarily Kupffer cells) in these animals do not express TLR4 (Fig 1A and 1B).

## Materials and Methods

### Animals

This study was carried out in strict accordance with the recommendations in the Guide for the Care and Use of Laboratory Animals of the National Institutes of Health. The protocol was approved by the Institutional Animal Care and Use Committee at the University of Illinois at Chicago (Protocol approval #12–152).

Six week old C57BL/6-Tg-(UBC-GFP) (TLR4+/+) and C57BL/10ScN (TLR4-/-) mice were purchased from Jackson Laboratories (Bar Harbor, ME). Green fluorescent protein (GFP) is under transcriptional control of the human ubiquitin C promoter resulting in high-level expression in most tissues.

Following one week acclimatization on regular chow and water *ad libitum* at a constant temperature (22°C) with humidity of 45% to 55% in a 12-hour light/dark cycle, all recipient mice for bone marrow transplant received water containing polymyxin 13mg/L (Sigma, St. Louis, MO), and neomycin 100mg/L (Sigma, St. Louis, MO) for 5 days. Mice were fed a low fat (LF) diet (12.3 kcal % fat, Research Diets, Inc., New Brunswick, NJ) (S1 Table) throughout the study.

### Generation of chimeric mice

After acclimatization and one week consumption of water with antibiotics, the following chimeras were made by adoptive bone marrow transplantation: C57BL/6-Tg-(UBC-GFP) (TLR4+/+):C57BL/6J (TLR4+/+); this chimera is designated as GFPWT:WT and C57BL/6-Tg-(UBC-GFP) (TLR4+/+):C57BL/10ScN (TLR4-/-); this chimera is designated as GFPWT:KO.

The chimeras were generated as follows: eight-week old recipient mice were irradiated using 9Gy. Bone marrow cells were isolated from the tibias and femurs of 6 week old donor mice (GFPWT) according to the methodology described by Saberi et al. [21]. Briefly, donor mice were sacrificed using CO_2_ gas, followed by cervical dislocation. Bone marrow was flushed from tibias and femurs into 50ml conical tubes with DMEM. Flushing was repeated three times and exudate mixed well to break up clumps using a serological pipette. This process was repeated 5 times, or until there were no large visible pieces of marrow in the mixture. The mixture was then put through a strainer (40μm) to remove bone chips and centrifuged at 950 RPM for 10 minutes at 4°C. After centrifugation, media was decanted and bone marrow cells resuspended in 10ml of DMEM with no FBS. The number of bone marrow cells was determined using a hemocytometer.

Approximately 5 million donor bone marrow derived cells (BMDC) were transplanted via the retro-orbital sinus of TLR4+/+ and TLR4-/- mice using a 27 ½ G needle attached to a 1 ml syringe (Becton Dickenson & CO, Franklin Lakes, New Jersey). Chimeric mice were maintained on water containing antibiotics (polymyxin 13mg/L [Sigma, St. Louis, MO] and neomycin 100mg/L [Sigma, St. Louis, MO]) for 5 weeks. Food intake and body weight were measured twice a week and well-being was checked twice daily following bone marrow transplantation.

### Confirmation of bone marrow transplantation

The success of bone marrow transplantation was confirmed by collecting circulating blood from chimeras and performing flow cytometric analyses to detect GFP positive cells. Briefly, 100μl of tail blood was collected in heparinized capillary tubes, RBCs were lysed using RBC lysis buffer (420301) (BioLegend, San Diego, CA) and the remaining cells were washed twice with PBS. Propidimum iodide (10 mg/ml) was added 10 min before flow cytometric analysis to detect dead cells.

### Study design

**Fig 2:**
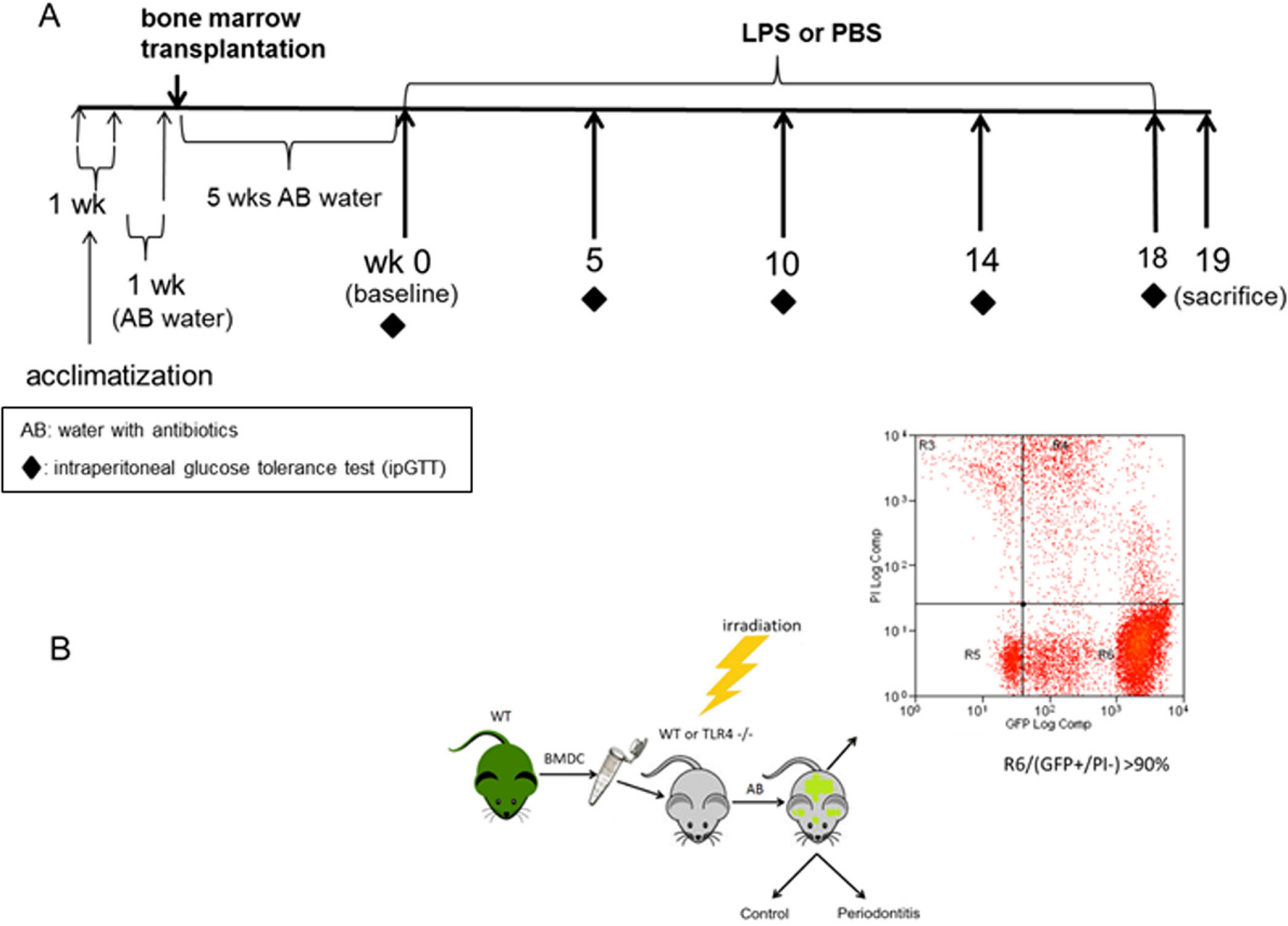
Schematic illustration of the experimental timeline and generation of chimeric mice. (A) Timeline, (B) Generation of chimeric mice: Bone marrow derived cells (BMDC) were isolated from a donor mouse (GFPWT) and transplanted to an irradiated recipient mouse (either WT or TLR4-/-). Following 5 weeks of consumption of water with antibiotics (AB), peripheral blood was drawn and FACS analysis performed confirming that >90% of circulating blood cells expressed GFP.

Five weeks following chimera formation, animals were divided into 4 groups: GFPWT:WT with LPS or PBS application which is designated as periodontitis (P) or control (C) respectively and GFPWT:KO with P or C. Periodontitis was induced by applying E. coli LPS 0111:B4 (10μg/μl, 1μl/site, Sigma, St. Louis, MO) to the palatal gingival sulcus of maxillary second molars [22,23] on Monday, Wednesday, and Friday every week for 18 weeks (Fig 2). PBS was applied in C animals instead of LPS. LPS or PBS was applied after animals were weighed and anesthetized using isoflurane. Mice were sacrificed during the 19^th^ week (24 weeks post-transplantation) by CO_2_ inhalation and cervical dislocation. A portion of liver from each animal was snap frozen in Trizol. One half of each maxilla was harvested and stored in 10% formaldehyde for 24 hrs, rinsed with PBS 3X, placed in 70% ethanol and shipped to the College of Veterinary Medicine, Veterinary Diagnostic Laboratory at the University of Illinois Urbana-Champaign for decalcification and paraffin embedding. The other half of each maxilla was used to assess bone loss by microtomography by an investigator (LL) who was blinded to the group to which the animal belonged.

### Immunofluorescence microscopy

For each chimera, TLR4 and ED-1 were detected by immunofluorescence in sections from the maxilla and the liver as described by Li et al. [24]. Liver sections were permeabilized by incubating 30 min with 0.25% Tween 20 (170–6531) (BioRad, Hercules, CA) in PBS. Sections were then incubated either with rabbit anti-mouse TLR4 antibody (ab13556)(Abcam, Cambridge, MA) as primary at 1:100 dilution for 1.5 hrs, and donkey anti-rabbit IgG conjugated Alexa Fluor 594 at 1:800 dilution for 1 hr (A21207) (Invitrogen, Grand Island, NY) as secondary antibody, or with mouse anti-mouse ED-1 (anti-CD68) (ab31630) (Abcam, Cambridge, MA) at 1:100 dilution overnight at 4°C and donkey anti-mouse IgG (A10035) (Invitrogen, Grand Island, NY) conjugated with Alexa Flour 350 at 1:800 for 1 hr at room temperature. Isotypic controls were used to determine non-specific binding of antibodies.

The following primary antibodies were used at 1:100 dilution to detect cells producing IL1β, TNFα, IL6, MCP1, and MMP9 in the gingiva: rabbit polyclonal anti-IL1β (ab9722) (Abcam, Cambridge, MA), anti-TNFα (ab66579) (Abcam), anti-MCP1 sc-28879) (Santa Cruz Biotechnology, Dallas, TX), anti-MMP9 antibody ab124513) (Abcam). The secondary antibody was donkey anti-rabbit IgG conjugated to Alexa Fluor 568 (Invitrogen, Grand Island, NY) and used at dilution of 1:800. Positive cells were counted in 5 random fields visualized by immunofluorescence microscopy at 400 X magnification. The average count of cytokine positive cells per field was tabulated per group. Scoring was done by an investigator who was blinded to the sample group.

### Microtomography

To assess alveolar bone loss, image acquisition was performed at the Integrated Small Animal Imaging Research Resource (iSAIRR) at the University of Chicago, Chicago, IL. Maxillae were scanned on a GMI Triumph with the following parameters: voltage: 60k; V current: 140 mA; field of view: 28.16 mm; projections: 1024; pixel size: 54 mm. AMIRA software (FEI, Hillsboro, OR) was used for image analysis. Maxillas were rendered in 3D and cross sections cut through the middle of the second molar were visualized. Linear measurements were taken in millimeters from the cemento-enamel junction (CEJ) to the alveolar bone crest to assess bone loss.

### Intraperiotoneal glucose tolerance test (ipGTT)

ipGTTs were performed at baseline (5 weeks post bone marrow transplantation), and at 5, 10, 14 and 18 weeks post-initiation of LPS/PBS treatment. Briefly, following a 14 hr fast and collection of blood samples to determine glucose and insulin levels, 50% dextrose (2 g/kg body weight) was administered intraperitoneally and glucose levels in tail blood were determined after 15, 30, 60, 90 and 120 min using a glucometer.

### Plasma cytokine and serum LPS determination

Whole blood was collected from the retro-orbital plexus at week18 and plasma was sent for the analysis of IL-1β, IL-6, and TNFα concentrations (Quansys Biosciences, Logan, Utah). Whole blood was collected from the retro-orbital plexus 24 hrs after the last LPS/PBS oral application at week 18. Serum was isolated and diluted at 1:10 and the concentration of LPS determined using Endpoint Chromogenic LAL Assay (Lonza, Allendale, NJ).

### Real time RT-PCR for liver expression of TLR4, F4/80, IL1β, IL6 and TNFα

Expression levels of TLR4, the macrophage marker F4/80, and proinflammatory cytokines IL1β, TNFα and IL6, which are known to interfere with insulin signaling in the liver, were determined by qRT-PCR.

Briefly, total RNA was extracted from 50 mg of frozen liver samples using TRIzol reagent (Invitrogen Corp., Carlsbad, CA) according to the manufacturer’s instructions.

Complementary DNA (cDNA) was synthesized using an Applied Biosystems High Capacity cDNA Reverse Transcription Kit (Applied Biosystems/Ambion) and real-time PCR was performed with cDNA from 25 ng of total RNA/sample in a 20 μL final volume using a TaqMan Gene Expression Master Mix and StepOne Plus Real Time PCR System (Applied Biosystems, Austin, TX, USA). The primers used were for: TLR4 (Mm00445273-m1) (Life Technologies, Grand Island, NY 14072), F4/80 (Mm00802529-m1) (Life Technologies, Grand Island, NY 14072), mouse IL-1β (Mm01336189m1) (Applied Biosystems), TNF-α (Mm99999068m1) (Applied Biosystems), and IL6 (Mm00446190m1) (Applied Biosystems). Each sample was run in duplicate and the mean value of each set of duplicates was normalized to mouse β-actin and used to calculate relative gene expression by the ΔΔCt method.

### Statistical analysis

Statistical analyses were performed using a Mann-Whitney test for insulin and glucose levels, HOMA, and ELISA. Student’s t-test was used for qPCR and LPS analyses. p<0.05 was considered statistically significant for all analyses.

## Results

### Confirmation of successful bone marrow transplantation

The success of bone marrow transplantation was confirmed by determining the % of GFP+ cells in blood 5 weeks following the transplant by fluorescence activated cell sorting (FACS) analysis. As evidenced in Fig 2B, greater than 90% of circulating white blood cells express GFP.

**Fig 3:**
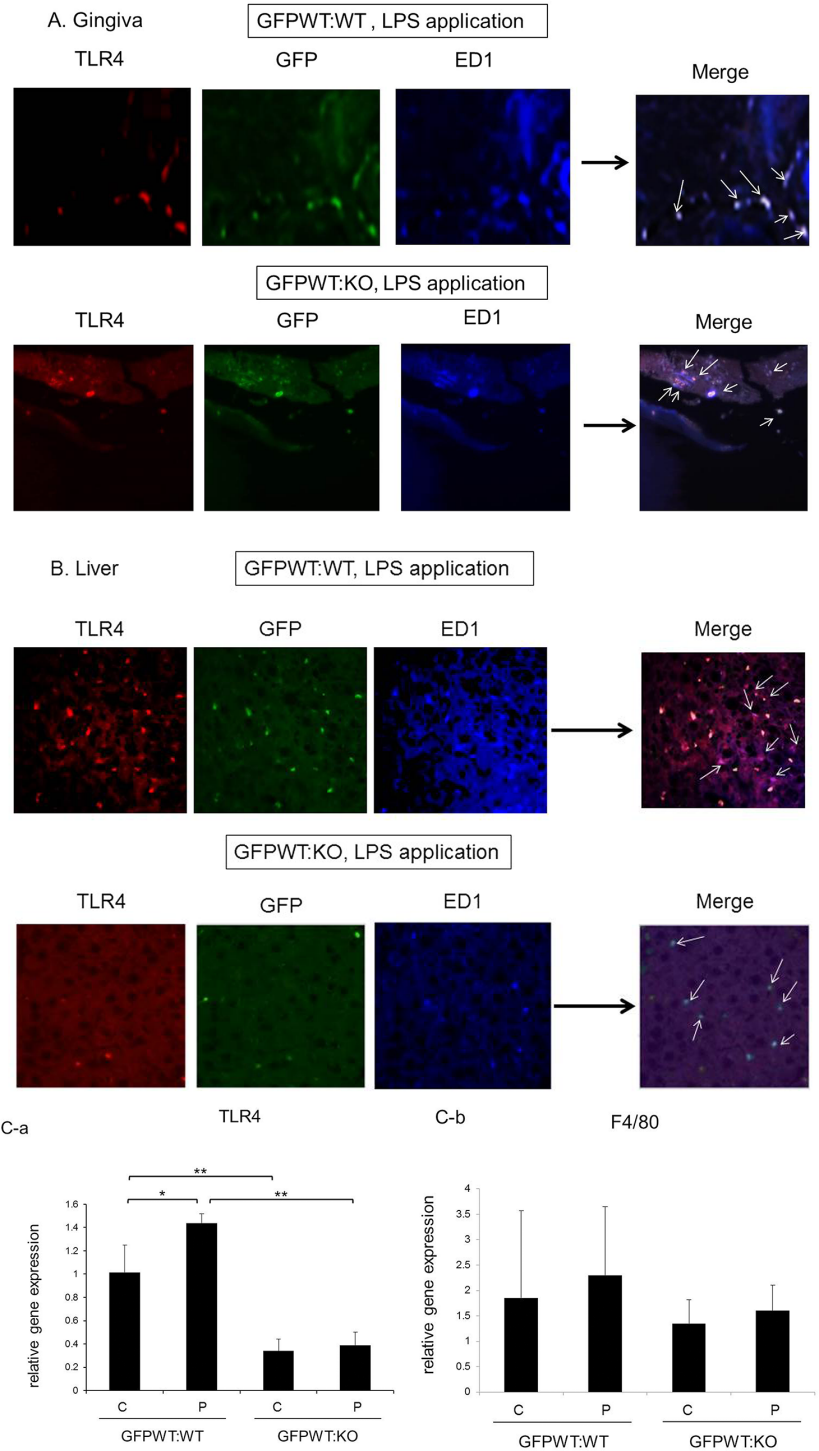
Further confirmation of chimera generation by immunofluorescence microscopy and qPCR. Donor macrophages migrate into the gingiva of both GFPWT:WT and GFPWT:KO mice, while Kupffer cells retain their recipient phenotype. (A) Immunofluorescence microscopy identifying TLR4+ macrophages (ED1+) in gingival tissue adjacent to a tooth in GFPWT:WT (top panel) and GFPWT:KO (bottom panel) chimeras following LPS application. Gingival macrophages in both GFPWT:WT and GFPWT:KO chimeras express TLR4+, GFP+, and ED1+ following LPS application. Red: TLR4+, Green: GFP+, Blue: ED1+. Arrows indicate migrating donor macrophages. (B) Immunofluorescence microscopy identifying liver Kupffer cells maintaining recipient’s phonotype in a GFPWT:WT chimera following LPS application (top panel, arrows: TLR4+, GFP-, ED1+ cells). Kupffer cells in GFPWT:KO are TLR4-, GFP-, ED1+ (bottom panel, arrows: TLR4-, GFP-, ED1+ cells). The few GFP+,TLR4+, ED1+ cells are likely migrating donor macrophages replacing recipient Kupffer cells. Red: TLR4+, Green: GFP+, Blue: ED1+. (C) Real time PCR analysis of liver TLR4 (a) and F4/80 (b) expression. Liver expression of TLR4 and the macrophage marker F4/80 was analyzed by TaqMan Real Time PCR. TLR4 expression was significantly higher in GFPWT:WT animals compared to GFPWT:KO mice (a). There is no statistical difference in F4/80 expression between GFPWT:WT and GFPWT:KO mice. x-axis: chimeric group, y-axis: relative expression of TLR4 (a) and F4/80 (b). All normalized to β-actin. n = 8–12 per chimeric group (4–6 per treatment group). Data presented are mean ± SD. *p<0.05, **p<0.001.

Immunofluorescence microscopy of tissue samples from GFP:WT and GFPWT:KO chimeras following application revealed that gingiva in both chimeras express TLR4+, GFP+ and ED-1+ cells (migrating gingival macrophages originated from BMDC) (Fig 3A). However, the liver of GFPWT:KO chimeras exhibited very few TLR4 positive cells (Fig 3B). The few TLR4+, GFP+, ED1+ cells in the liver of GFPWT:KO mice were likely donor macrophages replacing liver Kupffer cells.

Liver expression of TLR4 and the macrophage marker F4/80 was analyzed by TaqMan Real Time PCR (Fig 3C). Expression of TLR4 in the liver was significantly less in GFPWT:KO chimeras (Fig 3C-a). There is no significant difference in the expression of F4/80 among chimeric groups, although the mean expression level is higher in GFPWT:WT chimeras (p>0.5) (Fig 3C-b).

### The serum concentration of LPS was significantly higher in both chimeras with LPS application compared to chimeras PBS application

**Fig 4:**
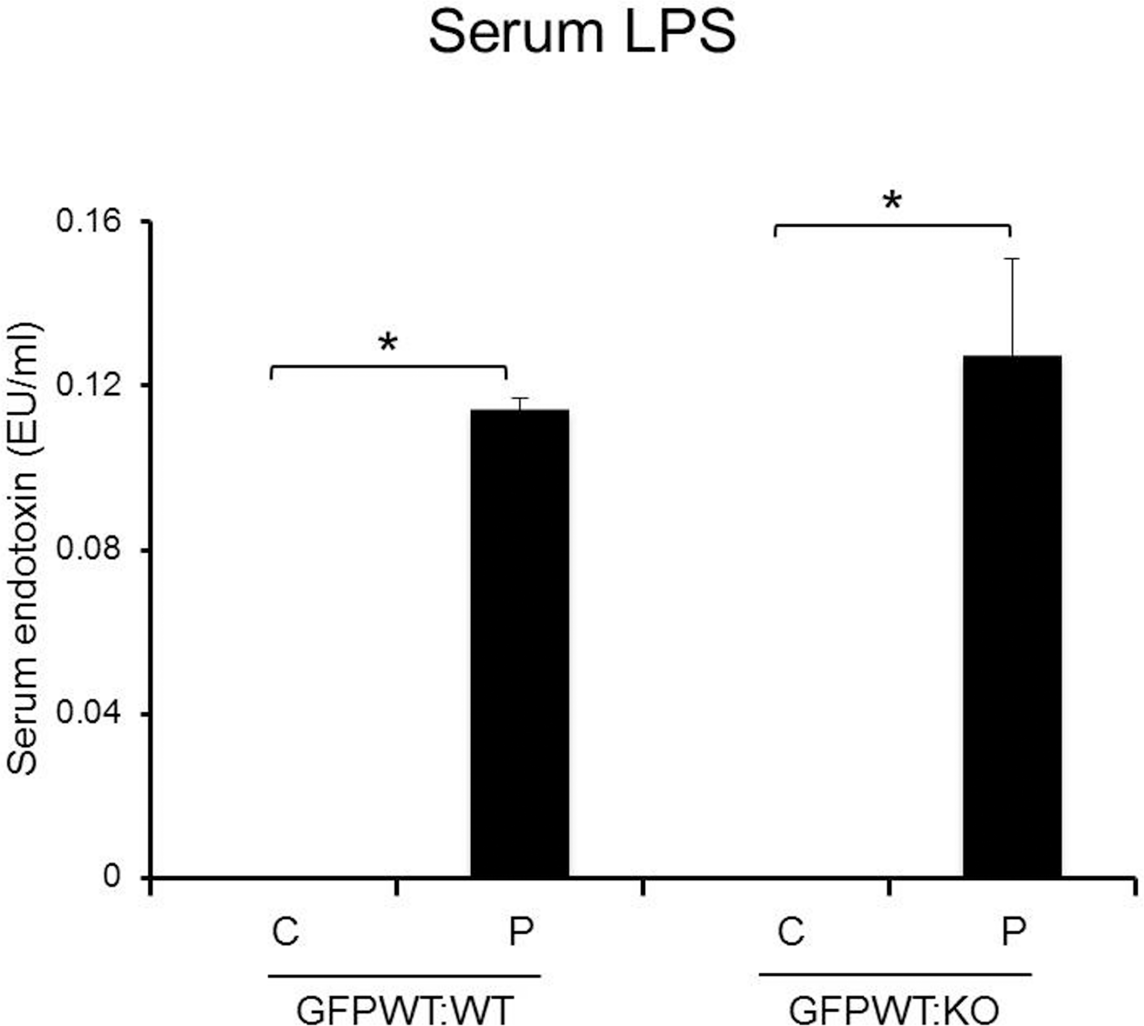
Serum LPS concentration is determined in animals with LPS vs PBS application. LPS is present only in the plasma of animals treated with LPS. x-axis: chimeric group, y-axis: LPS concentration (EU/ml). Data presented are mean ± SD. n = 8–12 per chimeric group (4–6 per treatment group). Data presented are mean ± SD. *p<0.001

The mean concentration of LPS was 0.11–0.13 EU/ml in GFPWT:WT and GFPWT:KO chimeras respectively after repeated application of LPS for 18 weeks. The chimeras with PBS treatment had no detectable LPS (Fig 4). This suggests that some of the LPS applied to gingival sulci reached the systemic circulation and even after 24 hrs of LPS application to gingival sulci, LPS was still in the systemic circulation in this chronic periodontitis model system.

### LPS application to gingival sulci induced gingival inflammation and bone loss

**Fig 5:**
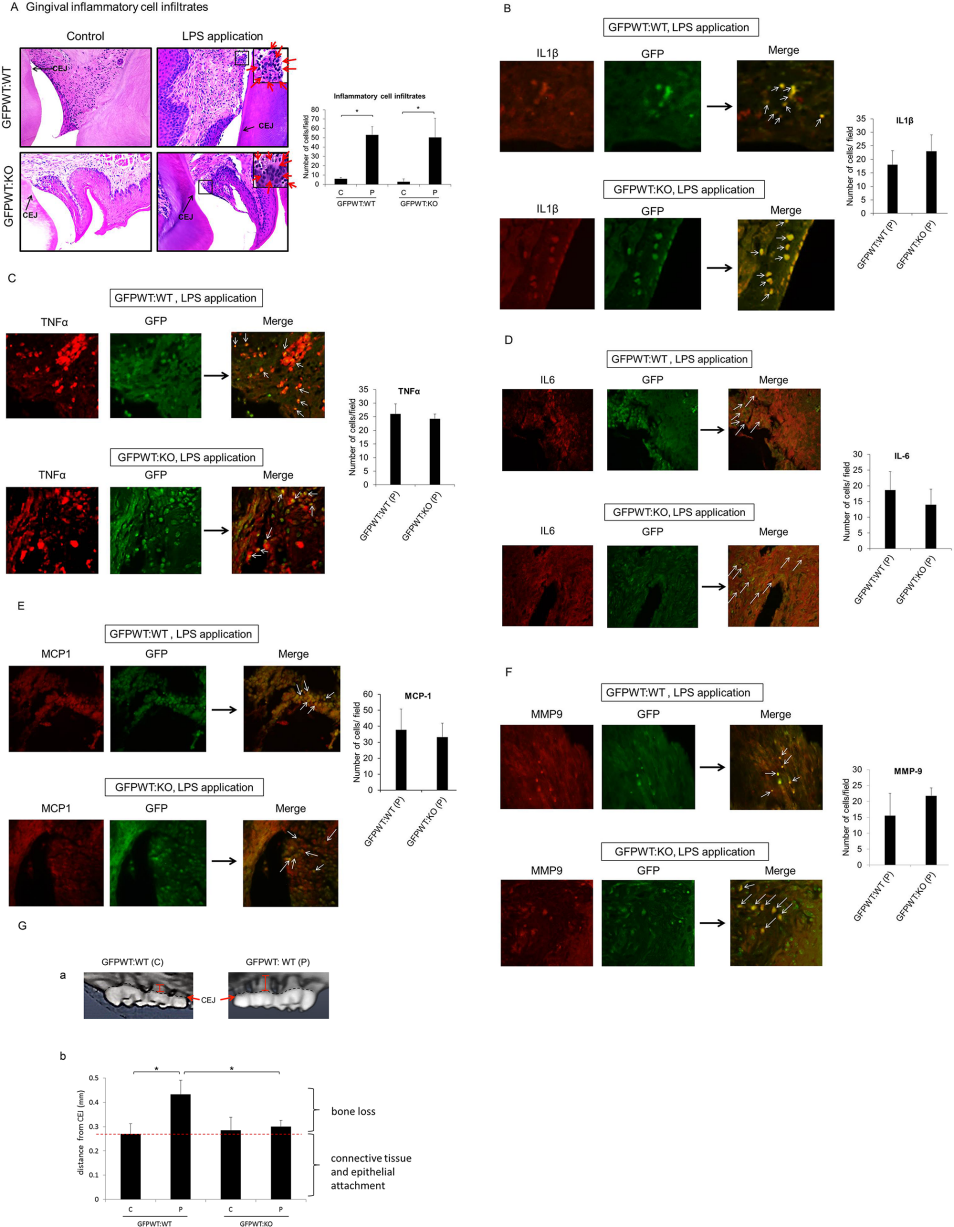
LPS application to gingival sulci induced gingival inflammation and bone loss. Gingival inflammation was present in both chimeras after gingival application of LPS (A-F). The degree of gingival inflammation was confirmed by the number of inflammatory infiltrates (A) and identification of IL1β (B), TNFα (C), IL6 (D), MCP1 (E), and MMP9 (F) producing cells. Inflammatory infiltrates were quantitatively measured using sections stained with H&E (n = 4 per treatment group) (A). Number of cells expressing cytokines (B, C, D), MCP1(E), and MMP9 (F) was assessed by immunofluorescence microscopy (n = 4 per treatment group). Inflammatory infiltrates were present in both GFPWT:WT and GFPWT:KO mice treated with LPS (A). Insets show inflammatory cell infiltrates. Arrows point to inflammatory cells. The number of inflammatory cells was significantly higher in LPS treated animals compared to PBS treated animals in both chimeric groups (p< 0.01) (A). There was no statistical difference in the amount of infiltrates between these chimeras with LPS application (A). There was also no statistical difference in the number of cells stained with IL1β, TNFα, IL6, MCP1 and MMP9 (B-F) between GFPWT:WT and GFPWT:KO mice with LPS application. Arrows indicate GFP+ donor cells expressing IL1β (B), TNFa (C), IL6 (D), MCP1 (E), and MMP9 (F) respectively. Inflammatory cell infiltrates were counted in 5 random fields visualized by light microscopy at 400X magnification. Photos of H&E sections were taken at 200X magnification. Insets are at 400X (A). Cells positive for cytokines, MCP1 and MMP9 were counted in 5 random fields and visualized by immunofluorescence microscopy at 400X magnification. Photos of immunofluorescence microscopy were taken at 400X (B-F). In all cases, the examiner was blinded to the animal group to which the tissue section belonged. x-axis: chimeric group. y-axis: number of cells/random field. Data presented are mean ± SD. (G) Alveolar bone loss was assessed by microtomography: Microtomography of maxilla from GFPWT:WT chimeras following 18 weeks of LPS or PBS treatment. The distances between the cemento-enamel junction (CEJ) and the alveolar crest are shown in red. The distance between the CEJ and the alveolar bone shown in a GFPWT:WT chimera without LPS application is occupied by junctional epithelium and connective tissue attached to the root and therefore does not reflect actual bone loss (G-a). Comparison of bone loss among chimeric groups (G-b):The bracket on the right bottom indicates the mean distance where epithelia and connective tissue attach. The bracket on the top indicates the mean bone loss. Bone loss was statistically greater in GFPWT:WT mice with LPS compared to PBS treatment. There is no statistically significant difference in bone loss between PBS and LPS treated groups in GFPWT:KO animals (G-b). x-axis: chimeric group, y-axis: distance (mm) measured from CEJ to the crest of alveolar bone on the mid palatal portion of the second maxillary molars. n = 8–12 per chimeric group (4–6 per treatment group). Data presented are mean ± SD. *p<0.05.

The degree of gingival inflammation was determined in both chimeras by assessing the number of inflammatory cell infiltrates as well as cells producing IL1β, TNFα, IL6, MCP1 and MMP9 (Fig 5A–5F). There was a significant amount of inflammatory cell infiltrates in both chimeras with LPS application compared to controls (Fig 5A). However, no significant difference was detected in the number of infiltrates between GFPWT:WT and GFPWT:KO with LPS application. Furthermore, there were no significant differences in the number of cells expressing the aforementioned proinflammatory cytokines or MCP1 and MMP9 between GFPWT:WT and GFPWT:KO that were treated with LPS.

As determined by microtomography, there was significantly more bone loss as a result of repeated E. coli LPS applications to the gingival sulcus in GFPWT:WT chimeras compared with GFPWT:KO chimeras (p<0.05) and GFPWT:WT chimeras treated with PBS (Fig 5G-a). The distance between the CEJ and the alveolar crest shown in GFPWT:WT chimeras without LPS application is the area where junctional epithelium and supra-alveolar connective tissue attach to the root at and just apical to the CEJ. Therefore, this is not the actual bone loss. Bone loss is minimal in GFPWT:KO chimeras with LPS application (Fig 5G-b).

### Impaired glucose tolerance occurs in GFPWT:WT chimeras with periodontitis, but not in the GFPWT:KO chimeras

**Fig 6:**
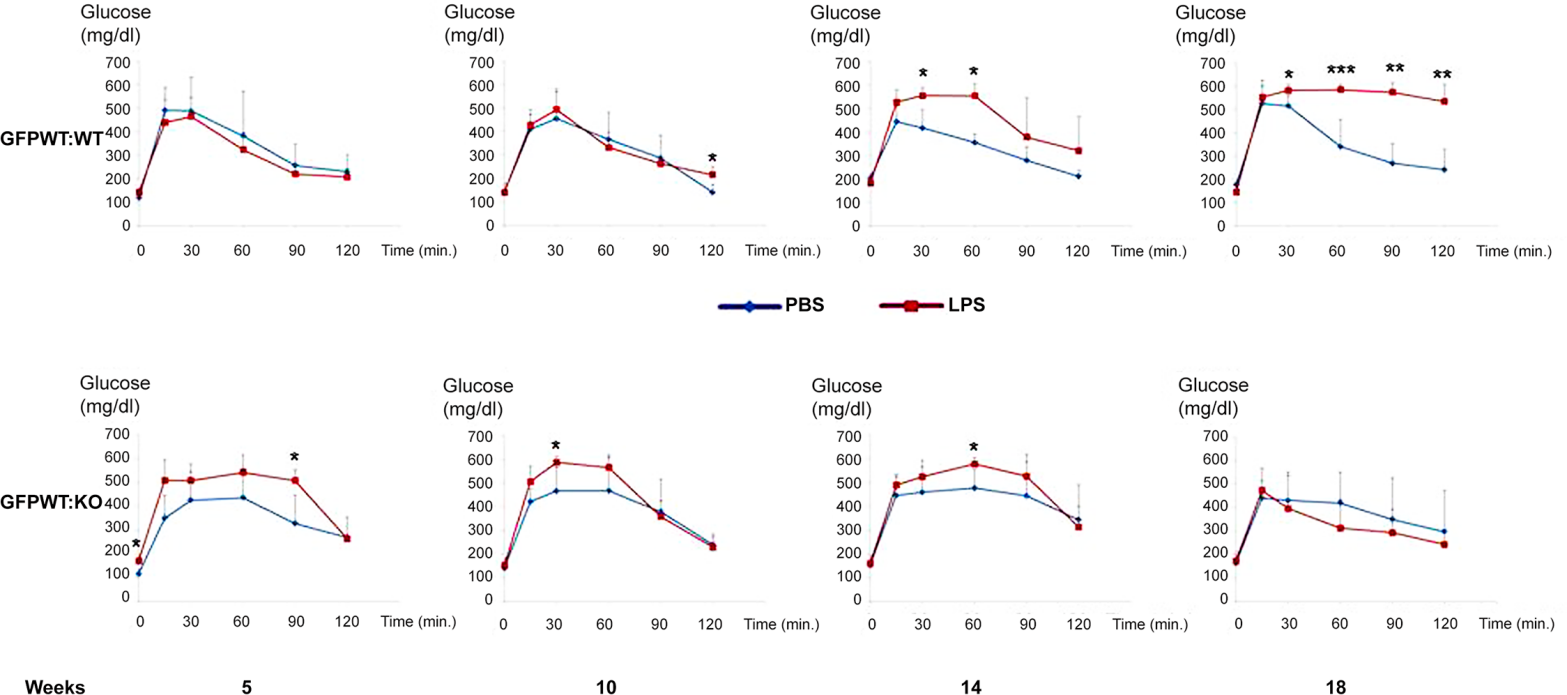
Glucose tolerance test results over 18 weeks. Severe glucose intolerance developed by 18^th^ week in GFPWT:WT animals with periodontitis. ipGTT was performed at week 0 (baseline), 5, 10, 14 and 18 weeks in GFPWT:WT (upper panel) and GFPWT:KO chimeras (lower panel) with LPS or PBS application. There was no difference in ipGTT results between these chimeras at baseline (data not shown). Data presented at each time point are mean ± SD. x-axis: post dextrose injection in min, y-axis: glucose levels (mg/dL). n = 8–12 per chimeric group (4–6 per treatment group). *p< 0.05, **p< 0.01, ***p< 0.001.

Glucose tolerance was monitored every 4–5 weeks following the establishment of chimeras (Fig 6). There was no difference in ipGTT between GFPWT:WT and GFPWT:KO chimeras at baseline (data not shown). Glucose tolerance was impaired at 30 and 60 min post-glucose treatment in GFPWT:WT chimeras after 14 weeks of LPS application and at 30, 60, 90, and 120 min after 18 weeks of LPS (Figs 6 and S1). In contrast, glucose tolerance was largely unchanged in GFPWT:KO mice treated with LPS vs PBS for either 14 or 18 weeks. ipGTT remained within the normal range during the 18 week period in GFPWT:WT chimeras with PBS application. Thus, in general, the application of LPS to gingival sulci did not cause significant differences in ipGTT in GFPWT:KO chimeras with and without LPS application. This indicates that the phenotype of TLR4 in recipient mice plays an important role in determining glucose tolerance. This further suggests that TLR4 activation in resident liver cells by LPS may play a primary and necessary role in determining glucose tolerance.

### Differences in fasting plasma glucose levels occur in weeks 5 and 18 in GFPWT:KO chimeras

**Fig 7:**
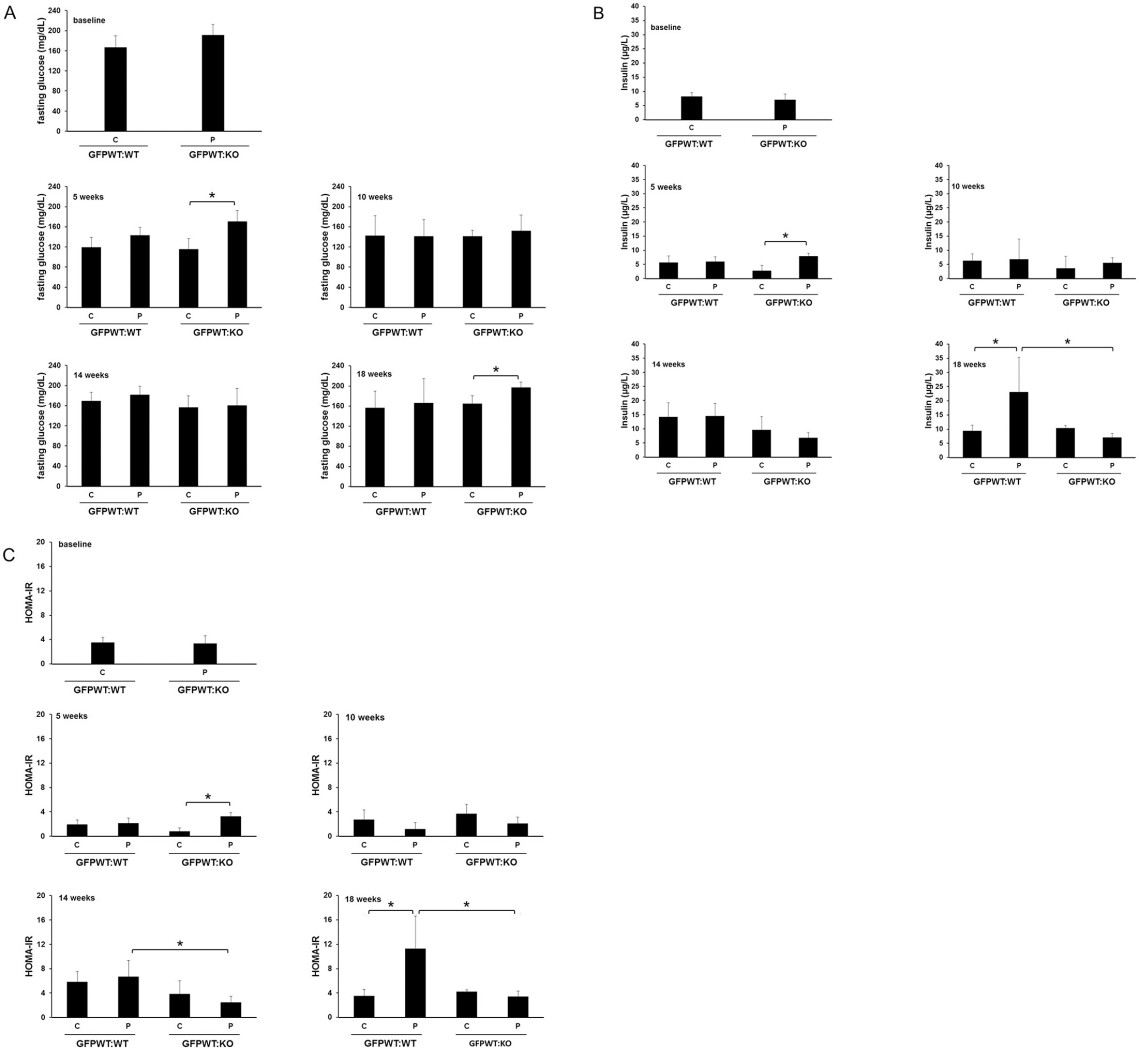
Fasting glucose, insulin and HOMA-IR measured over 18 weeks. Fasting glucose (A), insulin (B) and HOMA-IR (C) were measured at baseline and week 5, 10, 14 and 18. IR and fasting insulin levels were significantly higher in GFPWT:WT animals with LPS compared to PBS application at 18 weeks. x-axis: chimeric groups; GFPWT:WT, GFPWTKO, C: control (PBS application), P: periodontitis (LPS application). y-axis: fasting glucose concentration (mg/dL) (A), fasting insulin concentration(μg/L) (B), and HOMA-IR index (C). Data presented are mean ± SD. n = 8–12 per chimeric group (4–6 per treatment group). * p<0.05.

There were no statistically significant differences in plasma glucose levels at baseline between GFPWT:WT and GFPWT:KO chimeras following an overnight 14 hr fast (Fig 7A). At week 5, GFPWT:KO chimeras with LPS application developed significantly higher glucose levels compared to GFPWT:KO and GFPWT:WT without LPS application. At week 10 and 14 weeks, there was no significant difference among the different groups. At 18 weeks, GFPWT:KO chimeras with LPS application had significantly higher glucose levels compared to GFPWT:KO chimeras with PBS application (Fig 7A).

### Fasting insulin levels increase in GFPWT:WT chimeras with periodontitis in 18 weeks

Plasma samples were used to determine fasting insulin levels using a multiplex enzyme-linked immunosorbent assay. There was no statistically significant difference in insulin levels at baseline and at week 10 between groups (Fig 7B). At week 5, the level of insulin was decreased in GFPWT:KO chimeras without LPS application and this resulted in a statistically significant difference in insulin levels relative to GFPWT:KO chimeras with LPS application. At 18 weeks, insulin levels were significantly increased in GFPWT:WT chimeras with periodontitis relative to control animals and GFPWT:KO animals treated with LPS (p< 0.05) (Fig 7B).

### Insulin resistance develops by the 18^th^ week in GFPWT:WT chimeras with periodontitis but not in GFPWT:WT chimeras without periodontitis or in GFPWT:KO chimeras

IR was calculated from plasma samples using the homeostasis model assessment (HOMA-IR), where IR = (fasting glucose [mmol/l] x fasting insulin [mU/l])/22.5. At week 5, GFPWT:KO chimeras with LPS application developed IR compared to GFPWT:KO chimeras without LPS application. However, this statistical difference appears to be in part due to lower IR observed in GFPWT:KO chimeras without LPS application. By the 18^th^ week, the GFPWT:WT chimeras with periodontitis developed a significantly higher IR compared to their control animals and GFPWT:KO chimeras (Fig 7C).

### Plasma cytokines levels

**Fig 8:**
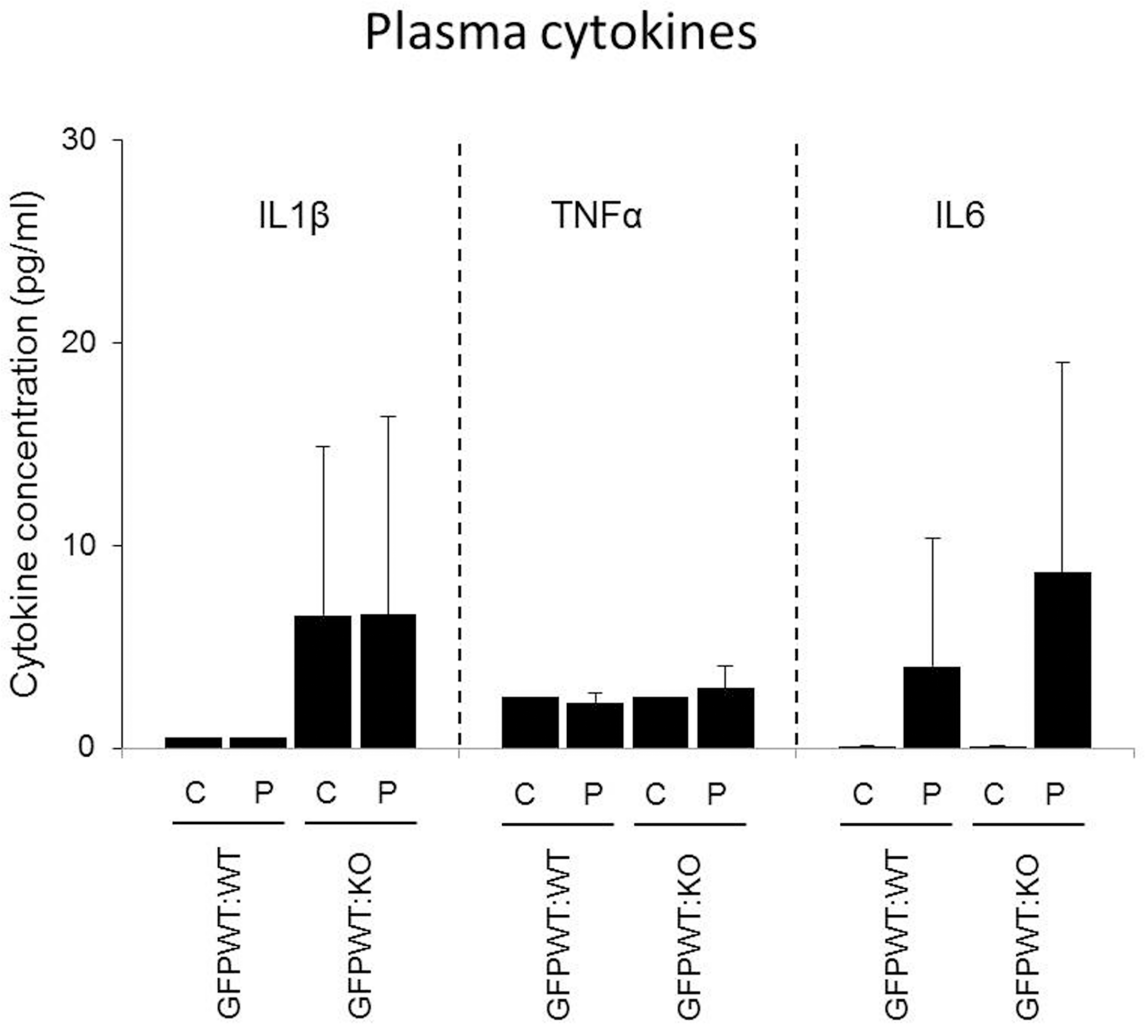
Plasma levels of cytokines measured by ELISA. Plasma levels of IL1β, TNFα, and IL6 which are known to cause IR were measured by Q-Plex ELISA. x-axis: chimeric group. y-axis: concentration in pg/ml. Data at 18 weeks presented are mean ± SD. There was no statistical difference among groups with either LPS or PBS application (p>0.05), n = 8–12 per chimeric group (4–6 per treatment group).

Levels of cytokines known to be involved in the development of IR were measured by ELISA (Fig 8). Although the mean levels plasma IL6 were higher in LPS vs PBS administered animals at 18 weeks, and IL1β was increased in both PBS and LPS treated GFPWT:KO groups relative to both GFPWT:WT groups, there were large standard deviations and no statistical differences between groups.

### Liver qPCR for TNFα, IL1β and IL6 expression

**Fig 9:**
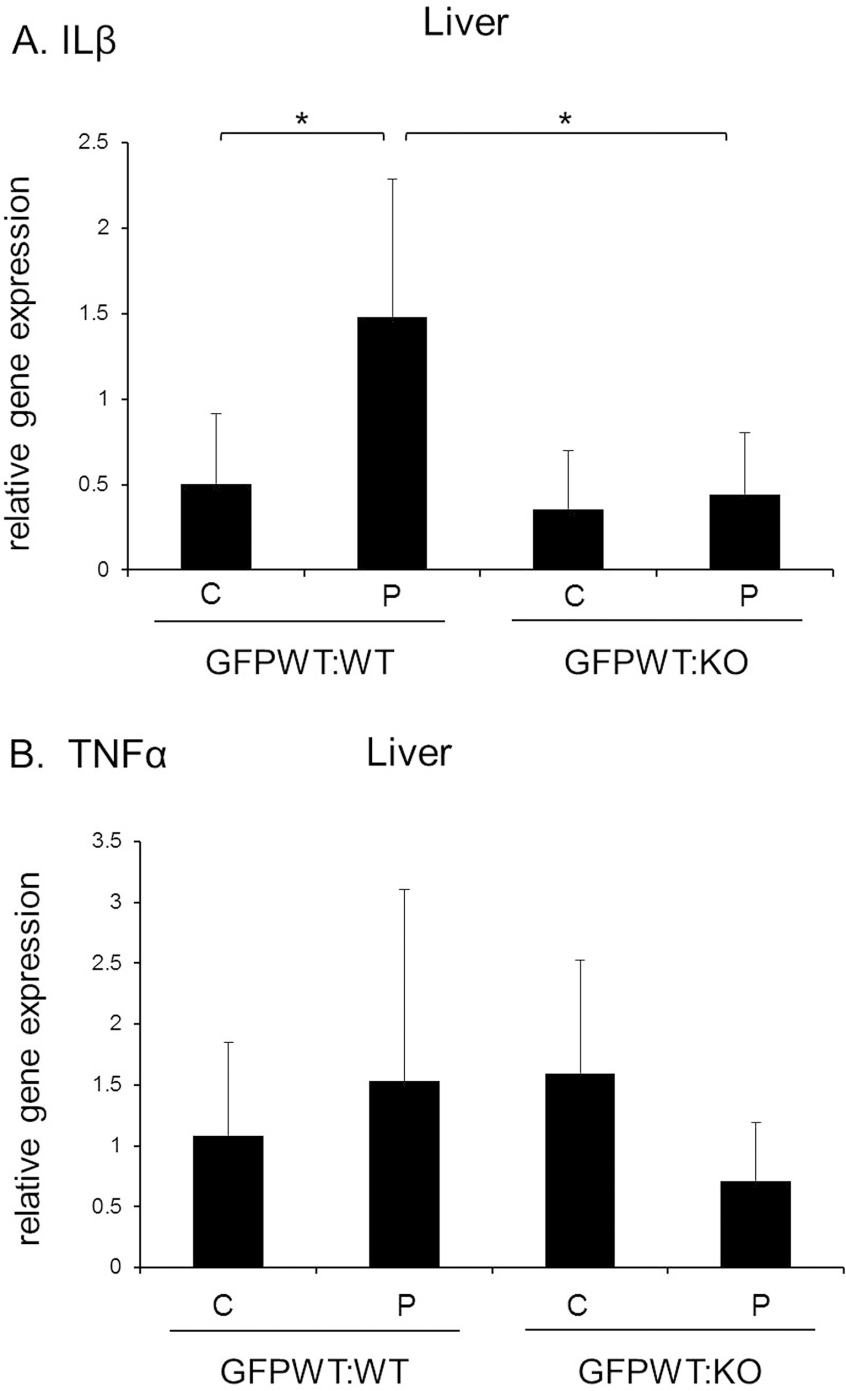
Expression of IL1β and TNFα in the liver determined by qPCR. (A) There was a significantly higher level of IL1β in GFPWT:WT mice treated with LPS compared to PBS treated animals and GFPWT:KO mice in both LPS and PBS treated groups. There was no significant increase in IL1β in animals treated with LPS or PBS in GFPWT:KO animals. (B) There was no significant difference in TNFα expression between chimeras with and without LPS application. IL-6 was not detected in the liver (data not shown). x-axis: chimeric group, y-axis: relative gene expression levels. Data presented are mean ± SD. n = 8–12 per chimeric group (4–6 per treatment group). *p<0.05.

The results of qPCR analysis of liver tissue indicated that liver IL1β expression was significantly higher in GFPWT:WT chimeras with periodontitis vs. no periodontitis at 18 weeks (p <0.05) (Fig 9A). However, there was no significant difference between GFPWT:KO chimeras with or without LPS application (Fig 9A). There was no significant difference in expression of TNFα between chimeras and with and without LPS application (Fig 9B). IL-6 expression was not detectable in the liver of any of the chimeras (data not shown).

### Body weight among groups over time

There were no statistically significant differences in body weight among groups over time (S2 Fig).

## Discussion

In spite of the commonly accepted concept that the relationship between periodontitis and diabetes is bidirectional, the mechanism(s) by which periodontitis impacts the development of prediabetes has not been identified.

The difficulty in determining the direct impact of periodontitis on the development of prediabetes is that periodontitis cannot be induced experimentally in humans to monitor its effect since it is an irreversible disease process. A few studies have investigated the influence of periodontitis on the development of GI/IR using various animal model systems [16,17,25,26]. The results from these studies led to the hypothesis that inflammatory cytokines from periodontitis sites influence the development of GI/IR. However, the precise mechanism(s) by which periodontitis influences GI/IR have not been elucidated and the chronic periodontitis/cytokine hypothesis has not been formally tested.

In our previous study, we investigated the effect of periodontitis on insulin signaling in the liver [17]. We showed that periodontitis impairs hepatic insulin signaling via TLR4 and that this impairment was less in mice with whole body TLR4 loss of function (LOF) when mice were fed a HF diet [17]. However, in this model system, the severity of periodontitis was much less in TLR4 LOF animals compared to TLR4 WT animals. Thus, it was not clear from these results if impaired insulin signaling was due to less severe periodontitis or due to an inability of TLR4 (LOF) animals to respond to periodontitis/LPS, particularly in the liver.

IR is a hallmark of prediabetes and can result from proinflammatory cytokines such as TNFα and IL1β causing impaired insulin signaling in insulin target organs [19,20]. TLR4 plays a major role in innate immunity by recognizing structural components of invading bacteria, such as LPS, and initiates host inflammatory responses to combat infections [27]. Binding of a TLR4 agonist to the receptor activates signal transduction via either MyD88 dependent or independent pathways and leads to secretion of proinflammatory cytokines such as TNFa, IL1β and IFNβ [18]. Among host cells which express TLR4, macrophages are known to express high levels of TLR4 and these cells in the gingiva when activated by LPS are known to produce pro-inflammatory cytokines [18,28]. Thus, macrophages play a key role in the initiation of host inflammatory responses via TLR4 activation by LPS. Since the primary etiological factor of periodontitis is predominantly gram negative bacteria and/or LPS, TLR4 expression by macrophages is critical in mediating host inflammatory responses in the gingiva by binding LPS. The liver contains 80–90% of all tissue/residential macrophages (Kupffer cells) in the body [29]. Kupffer cells which express high levels of TLR4 relative to other liver resident cells [30,31] are located in liver sinusoids, where they are the first cells exposed to enteric bacteria and LPS in the systemic circulation [32]. Therefore, bacterial byproducts can activate Kupffer cells via TLR4 [33–40] resulting in localized production of cytokines. Thus, if LPS from periodontal pathogens enters the systemic circulation, TLR4 expressed by Kupffer cells may play a key role in inducing hepatic insulin resistance [21].

To identify the relative involvement of migrating gingival and liver resident tissue macrophages, we generated chimeric mice which have WT TLR4 macrophages in the gingiva following LPS application but differ in that the liver resident macrophages do not express TLR4 in the GFPWT:KO chimeras. LPS acts as a chemoattractant and therefore macrophages migrate to the gingiva in both groups of chimeras. In commonly used procedures of creating a chimeric mouse, clodronate liposomes are used to eliminate liver Kupffer cells [41]. However, in our study, we wanted to retain TLR4-/- Kupffer cells in the recipient mice in GFPWT:KO chimeras and thus did not use clodronate.

We successfully generated a chimeric animal model system as evidenced by the presence of TLR4+ cells in the gingiva of both GFPWT:WT and GFPWT:KO chimeras and TLR4- cells in the liver in GFPWT:KO chimeras. The liver from GFPWT:KO chimeras exhibits few cells which express GFP based on immunofluorescence microscopy. It has been shown that approximately 30% of Kupffer cells are replaced in six months following bone marrow transplantation using irradiation but without the use of liposomal clodronate [42]. Thus, the few GFP positive cells in the liver of GFPWT:KO chimeras that co-express ED1+ may represent replenished Kupffer cells.

In these animals, we applied the TLR4 agonist, E coli LPS, three times a week for 18 weeks to mimic chronic periodontal infection/inflammation. Using these chimeras, we were able to induce chronic periodontitis which was validated by significant alveolar bone loss in GFPWT:WT chimeras with LPS local application to the gingival sulci but not with PBS application. In GFPWT:KO chimeras, the mean bone loss was only slightly higher in animals after gingival application of LPS compared with animals treated with PBS. This strongly suggests that TLR4 plays a major role in alveolar bone loss and that stimulation with the TLR4 pure agonist (E. coli LPS) alone is sufficient to cause severe bone loss in GFPWT:WT chimeras. However, it also suggests that recipient gingival resident cells that engage directly in bone resorption (osteoblast/osteoclast) may require TLR4 expression since GFPWT:KO chimeras with LPS treatment had no or minimal bone loss in spite of macrophage migration to the gingiva.

Although alveolar bone loss was not notable in the GFPWT:KO chimeras relative to GFPWT:WT treated with LPS, the GFPWT:WT and GFPWT:KO chimeras did not differ in regards to the degree of inflammatory cell infiltrates or cells expressing IL1β, TNFα, IL6, MCP-1, or MMP9 in response to LPS application. These data establish that LPS application result in gingival inflammation even when the preponderance of cells in the gingiva are TLR4-/- and that apparent bone loss may not be evident in this setting. The relative number of TLR4+/+ vs. TLR4-/- cells and the timing of inflammation initiation may be factors which influence the absolute level of inflammation and bone loss.

The serum concentration of LPS was significantly higher in the LPS application group compared to the control group in both chimeras, indicating that application of LPS to gingival sulci leads to higher systemic concentrations of LPS. This result is different from the study by Arimatsu et al., [43]. They have reported that there was no difference in LPS levels in fasting serum 16 hrs following oral application of 1 X 10^9^ CFU *P*. *gingivalis* (Pg) as compared to no Pg treatment controls although both groups had a detectable level of LPS. The difference in results may be due to the use of Pg oral application vs LPS application to the gingival sulci.

Using our model, we determined the development of IG and IR. We observed no significant differences in ipGTT in the GFPWT:KO chimeras with and without periodontitis, but there was a significant difference in ipGTT between GFPWT:WT chimeras with and without periodontitis. This indicates that the phenotype of TLR4, including Kupffer cells which are the predominant TLR4 expressing cells in the liver, plays a role in development of glucose intolerance. This further suggests that TLR4 activation of Kupffer cells in the liver may play a major role in determining the effect of periodontitis on glucose tolerance. Thus, although elevated levels of LPS are present in GFPWT:KO chimeras after LPS application, these animals do not develop IR or impaired ipGTT. This result suggests that LPS in the systemic circulation locally activates recipient cells in the liver, particularly Kupffer cells, via TLR4 which in turn activates production of IL1β which acts as an inhibitor of insulin signaling. The data suggest that periodontally produced cytokines have a limited effect on the liver in terms of GI/IR.

The level of IR was significantly higher in GFPWT:WT chimeras with periodontitis compared to all other groups: GFPWT:WT chimeras without periodontitis or GFPWT:KO chimeras with LPS or PBS application. The increased IR as calculated by HOMA was associated with increased fasting insulin levels rather than increased plasma glucose levels. An increased level of insulin observed in the WT animals with periodontitis is consistent with results from our previous studies [16,17]. The mechanism that leads to this increase in insulin is, however, not entirely clear. Recently, we have demonstrated that LPS isolated from the periodontal pathogen, Pg, stimulates insulin secretion from the pancreatic beta cell line MIN6 [44]. Thus it is possible that the increased plasma insulin is due in part to the effects of both hepatic insulin resistance and direct effects of LPS on insulin secretion.

The finding that plasma insulin levels were increased in GFPWT:WT chimeras with periodontitis and not in GFPWT:KO chimeras whereas LPS levels in plasma were elevated in both these chimeras with LPS application suggests that, in addition to its role in promoting IR in the liver, TLR4 may also play a role in promoting insulin secretion in periodontitis.

The levels of plasma cytokines TNFα, IL1β and IL6 at 18 weeks were not significantly different between LPS vs PBS application groups and also between chimeric groups. In contrast, analysis of expression of these cytokine genes in the liver indicated that IL1β was significantly higher in GFPWT:WT chimeras with LPS application compared to PBS application, or GFPWT:KO chimeras with or without LPS application. Since there was no difference in liver TNFα expression in these animals and IL6 was not detectable in the liver at 18 weeks, IL1β appears to be a key component in regulating insulin signaling, although it cannot be ruled out that other cytokines in combination with IL1β may have key roles.

The results from the present study indicate that TLR4 expression by liver resident cells plays a key role in translating the effects of chronic periodontitis into the development of insulin resistance and glucose intolerance. Since GI/IR is dampened in animals whose Kupffer cells are TLR4-, reducing TLR4 expression specific to Kupffer cells would appear to be beneficial. However, one of the major functions of Kupffer cells is to filter out enteric bacteria and LPS in the systemic circulation [45]. Thus, the proper approach to reduce GI/IR may be to counteract locally produced proinflammatory cytokines that are produced in response to LPS in the liver. Alternatively, inhibition of pathways which lead to the production of proinflammatory cytokines in Kupffer cells may be beneficial. These treatment approaches may inhibit the development of IR/GI triggered by chronic periodontitis, and hence may prevent the progression to frank diabetes.

To our knowledge, 18 weeks is the longest time for which mice have been exposed to a TLR4 agonist via gingival application and for which GI/IR were monitored. This study is the first to demonstrate the direct link and impact of LPS from periodontitis on the development of insulin resistance/glucose intolerance in the liver.

## Supporting Information

S1 Fig Area under the curve (AUC) measured using the trapezoidal rule. x-axis: chimeric group, y-axis: AUC (min x mg/dL). *p<0.01
(TIF)

S2 Fig Body weight among groups over time. x-axis: time (weeks), y-axis: body weight (g).
(TIF)

S1 Table Composition of diet (D12450B). (DOCX)
