# Supplementary figures and images for: TLR4 Expression by Liver Resident Cells Mediates the Development of Glucose Intolerance and Insulin Resistance in Experimental Periodontitis
Source: PLoS One. 2015 Aug 28;10(8):e0136502. doi: 10.1371/journal.pone.0136502 (PMC4552742; doi:10.1371/journal.pone.0136502)

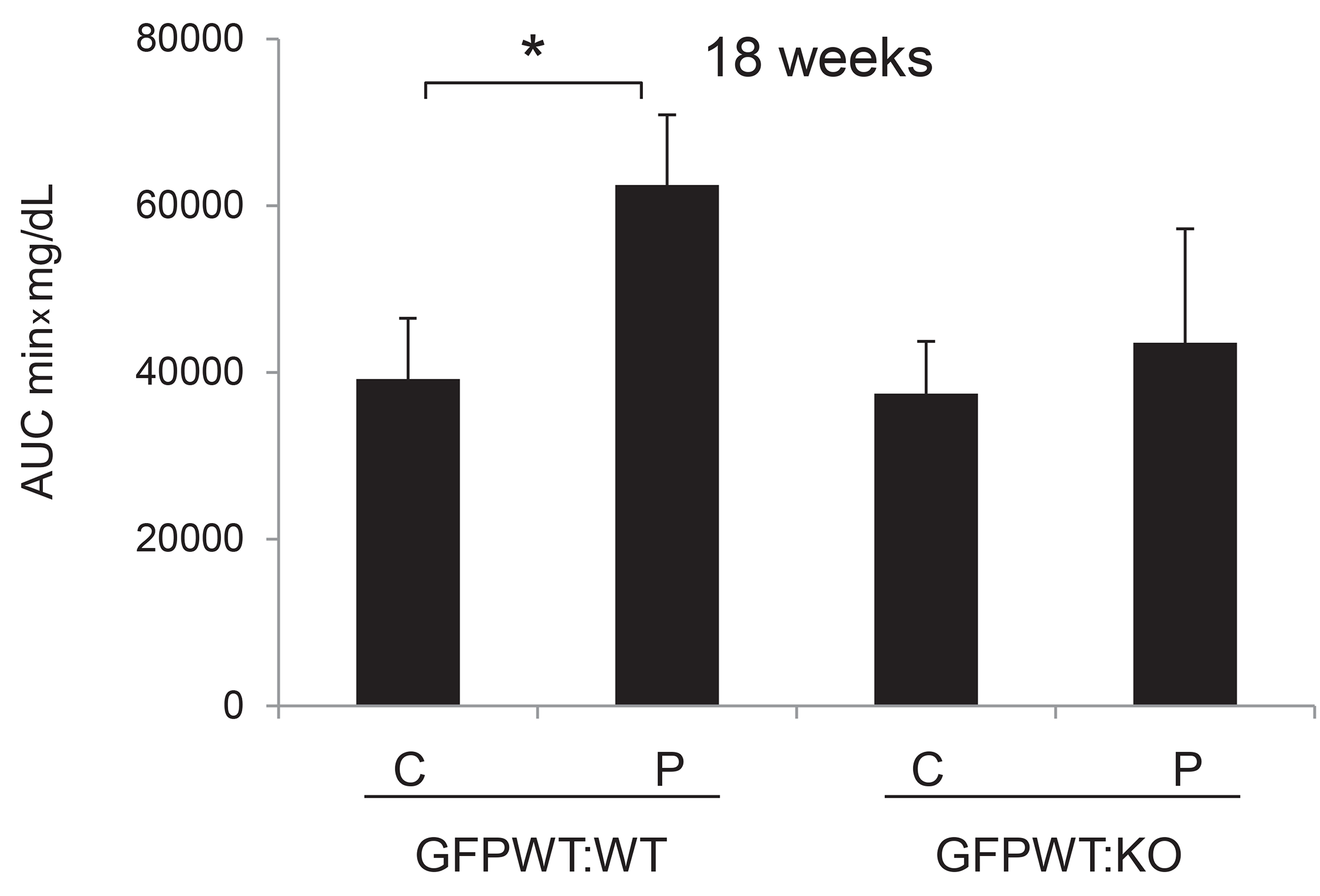

Supplement: S1 Fig — x-axis: chimeric group, y-axis: AUC (min x mg/dL). *p<0.01 (TIF) [file pone.0136502.s001.tif]

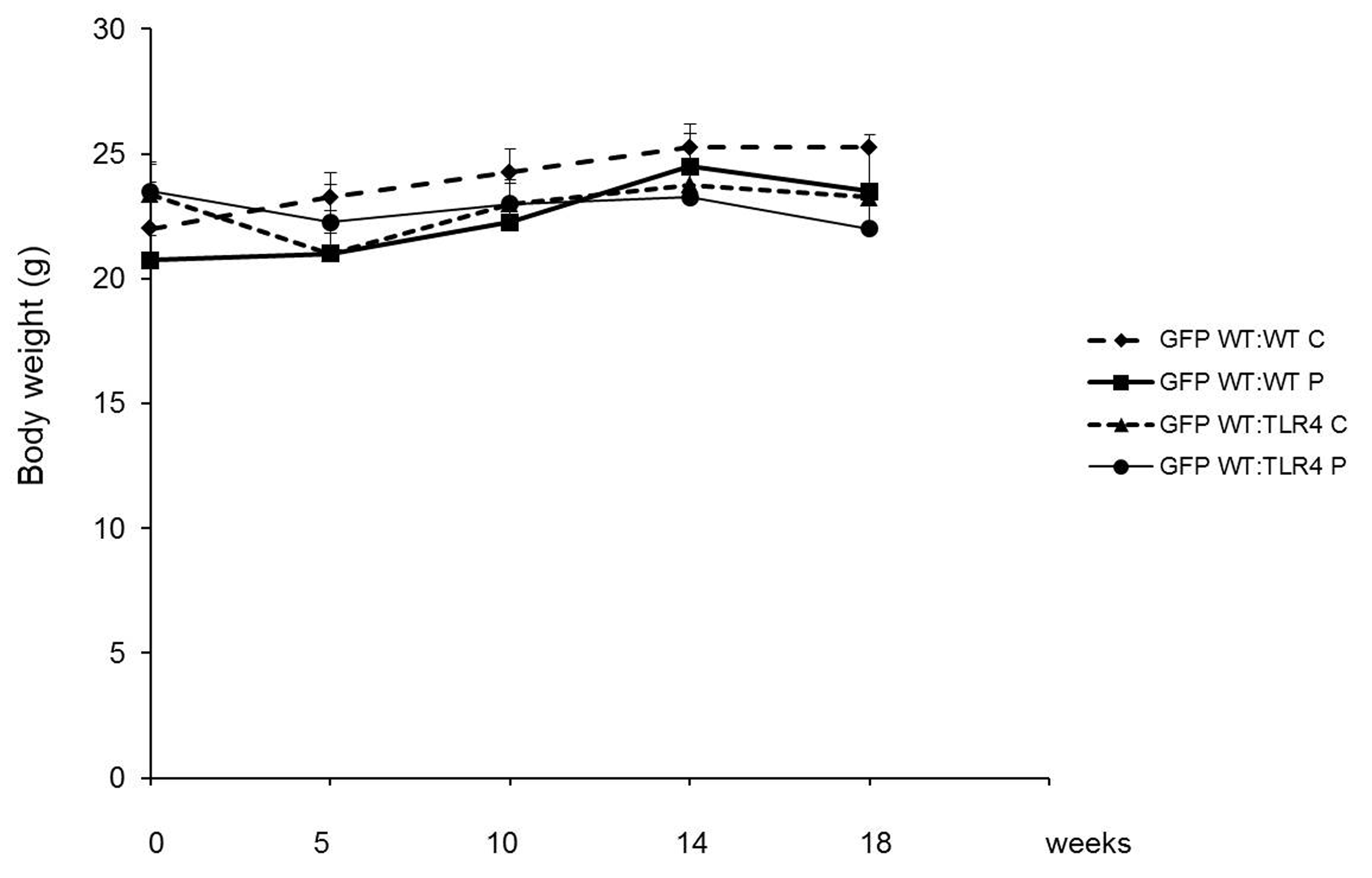

Supplement: S2 Fig — x-axis: time (weeks), y-axis: body weight (g). (TIF) [file pone.0136502.s002.tif]
