# Supplementary material for: TLR4 Expression by Liver Resident Cells Mediates the Development of Glucose Intolerance and Insulin Resistance in Experimental Periodontitis
Source: PLoS One. 2015 Aug 28;10(8):e0136502. doi: 10.1371/journal.pone.0136502 (PMC4552742; doi:10.1371/journal.pone.0136502)
Supplement: S1 Table — (DOCX) [file pone.0136502.s003.docx]

| **Composition of diet** | |  |  |
| --- | --- | --- | --- |
| **Ingredient** | | **gm** | **kcal** |
| Casein, 30 Mesh |  | 200 | 800 |
| L-Cystine |  | 3 | 12 |
| Corn Starch |  | 315 | 1260 |
| Maltodextrin 10 |  | 35 | 140 |
| Sucrose |  | 350 | 1400 |
| Cellulose, BW200 |  | 50 | 0 |
| Soybean Oil |  | 25 | 225 |
| Lard |  | 20 | 180 |
| Mineral Mix, S10026 |  | 10 | 0 |
| DiCalcium Phosphate |  | 13 | 0 |
| Calcium Carbonate |  | 5.5 | 0 |
| Potassium Citrate, 1 H2O |  | 16.5 | 0 |
| Vitamin Mix, V10001 |  | 10 | 40 |
| Choline Bitartrate |  | 2 | 0 |
| FD&C Yellow Dye #5 |  | 0.05 | 0 |
| **Total** |  | **1055.05** | **4057** |
